# Sublingual immunotherapy for allergy to shrimp: the nine-year clinical experience of a Midwest Allergy-Immunology practice

**DOI:** 10.1186/s13223-024-00895-7

**Published:** 2024-05-11

**Authors:** Lydia M. Theodoropoulou, Niamh A. Cullen

**Affiliations:** 1 Aquinas High School, La Crosse, WI USA; 2 Allergy Associates of La Crosse, Onalaska, WI USA

## Abstract

**Background:**

Diet restrictions and fear of adverse reactions put a significant burden on the nutrition, growth and life style of children and adults with food allergies. While various disease-modifying options are pursued, there are so far no published clinical data on immunotherapy for crustaceans. The efficacy and safety of desensitization to crustaceans by means of sublingual immunotherapy is assessed for the first time in this study with a view of validating it as a clinical-practice modality.

**Methods:**

Charts of a Midwest Allergy-Immunology practice from the period January 2014–June 2023 were reviewed to identify patients with allergy to shrimp treated with sublingual immunotherapy and to retrospectively evaluate their responses to oral challenge.

**Results:**

Sixty-six patients were identified who had been treated by sublingual immunotherapy for either systemic or localized reactions to shrimp. Demographics and relevant comorbidities were consistent with those of the atopic population. Sublingual immunotherapy with serially diluted mixtures was initiated at 64–320 ng/dose and was gradually escalated to 0.5 mg/dose three times a day. The sublingual immunotherapy course ranged from 5 to 72 months (average: 51 months), following which, 18 patients underwent shrimp oral challenge. No systemic reactions occurred upon challenge; no patient required epinephrine. Tolerance of target dose equal to or exceeding 42 g shrimp was achieved in 11 patients (61%), seven of whom had originally presented with systemic reactions to crustaceans. Seven patients (38%) developed one or more of the following localized reactions: oral itching, nasal symptoms, localized perioral hives, localized hives at pressure points, nausea, vomiting, abdominal pain upon exposure to a cumulative dose of 39.2–148.2 g of shrimp during the 4 h of the challenge. Five of these patients had originally presented with systemic reactions to crustaceans. Five of the 7 patients who developed localized symptoms during the challenge were subsequently placed on routine exposure to 12–20 g shrimp every other day. Two patients continued sublingual immunotherapy but declined routine exposure to shrimp every other day because they had no intention to incorporate crustaceans to their routine diet. On repeat challenge 6–9 months after original challenge, all five patients who had routine exposure to 12–20 g shrimp every other day tolerated the procedure to target dose without any symptoms.

**Conclusions:**

Desensitization to shrimp by sublingual immunotherapy appears to be safe and effective as shown in this study. Whether the immune modification induced by sublingual immunotherapy is permanent resulting in sustained tolerance, or the achieved degree of desensitization depends on regular exposure is not known; therefore, following challenge, regular consumption three-four times per week was recommended.

## Introduction

IgE-mediated food allergies affect 4–10% of children and 2–3% of adults [1]. After early childhood, permanent IgE-mediated food allergies are unlikely to spontaneously resolve, and their systemic manifestations upon exposure may lead to life-threatening events. Food allergies of systemic nature become a cause of anxiety for patients and their family affecting all aspects of life that pertain to food consumption [2]. Regarding shellfish, and shrimp in particular, anxiety is further heightened by the common catering practice to include shrimps as *hors d’oeuvres* and appetizers in social events where food is served, thus increasing the risk for contamination of all other foods offered.

There is no treatment that would eradicate a permanent allergy, and guidelines focus on prevention and management of accidental exposures with epinephrine, antihistamines, steroids, and other means to control systemic manifestations of acute mast cell activation and degranulation [2]. The clinical and pathological manifestations of the allergy status however can be altered by effective manipulation of immune responses to induce a level of tolerance [3]. In the past, such desensitization was attempted by means of subcutaneous immunotherapy with disappointing results [4]. It is for this reason that desensitization followed by subsequent continuous exposure to food allergen is now focusing on protocols of sublingual and/or oral administration [5, 6]. While a remarkable level of understanding and clinical applications has been attained in the induction of peanut tolerance, this is not the case with other foods [3, 5, 6]. Regarding allergy to crustaceans and desensitization treatment, practical approaches remain quite limited in spite of the fact that incidence of shellfish allergy is one of the most rapidly increasing food allergies, especially in the East Asian and Pacific populations [7, 8]. Immunotherapy for crustaceans is poorly researched and there are no standardized protocols for its implementation. The demand, however, for treatment is substantial, and several practitioners have developed genuine methods to treat allergy to crustaceans and eventually introduce to diet. As a result, many allergists have introduced to their practices desensitization protocols based on unpublished previous experience. Advanced basic research as well as wide-scale clinical trials are needed to delineate the nature of shellfish allergy and assess the sustainability of tolerance induced by immunotherapy. So far, no series or case reports of successful desensitization to crustaceans have been published following an immunotherapy protocol specific to crustaceans. There is, however, a case report of clinical improvement of symptoms upon exposure in a patient with shrimp allergy treated with sublingual immunotherapy tablets for house dust mite allergy. This report is consistent with the known cross-reactivity between shrimp and dust mite [9]. Herewith, desensitization to shrimp by means of sublingual immunotherapy is assessed for efficacy and safety with a view of validating it as a disease-modifying modality.

## Methods

Charts of a Midwest Allergy-Immunology practice from the period January 2014–June 2023 were reviewed to identify patients with allergy to crustaceans treated with sublingual immunotherapy and to retrospectively evaluate their responses to oral challenge. Patients with a presenting history of systemic or localized reactions to crustaceans were included based on history, positive IgEs to shrimp and positive skin tests to shrimp and other crustaceans. Patients with food allergies other than crustaceans were all included in the study if they had a history of systemic reactions to crustaceans. Patients who were not compliant with the immunotherapy protocol for at least 65% of doses, averaged per year of immunotherapy, were excluded. All patients had been previously evaluated and diagnosed with shrimp allergy by a Board certified Allergist-Immunologist.

Sublingual immunotherapy with five-fold serially diluted mixtures was to be initiated at 64 ng or 320 ng/dose (0.064 mg or 0.32 mg/dose). Commercially available extracts 50% v/v by Greer formulated for skin testing for *Farfantepenaeus aztecus* were used to prepare the serial dilutions used for immunotherapy. Initiating doses were empirically inversely titrated against IgE levels with the 64 ng dose assigned to patients who presented with shrimp IgE > 100 kU/L and the 320 ng dose for patients who presented with IgE < 10 kU/L. In-house IgE testing was with ImmunoCAP f24. Depending on availability and patients’ circumstances, doses were escalated gradually on variable intervals which ranged from weekly to quarterly, with an intention to reach maintenance dosing over a period of 6–48 months. Target dose was 0.5 mg/dose three times a day. Within six months of reaching target dose and while immunotherapy at this level was still going on, patients were to undergo shrimp oral challenge. Challenges were performed while patients were still receiving target-dose immunotherapy at 0.5 mg/dose.

Oral challenges by administration of gradually increasing doses were performed at the Allergy Associates of La Crosse–Challenges and Biologics Unit under continuous supervision by qualified personnel. Escalation of administered doses was every 20 min. Administration of each dose was preceded by vital signs, pulse oximetry and physical examination of eyes, ear-nose-throat, skin, respiratory and cardiovascular systems. Baseline spirometry was performed at the beginning of the challenge.

The initiating oral-challenge dose was 2 mg shrimp protein. This amount was administered in the form of 0.5 ml of 0.4% w/v shrimp-protein extract, which was diluted in glycerin/water solution to make up a volume of 10 ml. This step was followed by increasing doses of fresh, lightly boiled shrimp with a starting dose of 0.425 g. Boiling time was 45–60 s depending on size. Weight was expressed as the weight of peeled (shelled), deveined and lightly boiled shrimp (as opposed to shrimp protein, which is approximately 25% of a shrimp’s weight). Target dose was 42 g shrimp weight—approximately 4 medium-size shrimps. Cumulative amount at target dosing was 81.2 g of shrimp weight. Because of availability and convenient size, three shrimp species were used for the challenges: Farfantapenaeus aztecus (red shrimp), *Litopenaeus vannamei* (whiteleg sprimp) and *Acetes japonicus*.

Reactions to increasing doses were classified as localized versus systemic. Localized Reaction was defined as:

- one of any of the following: oral/peri-oral itching/numbness, peri-oral hives, hives limited to pressure points, nasal symptoms (congestion/runny nose/repetitive sneezing); or
- two symptoms if any one of the above was present and the second symptom was: malaise, dizziness, abdominal cramps, nausea/vomiting, anxiety, palpitations, provided that no hypotension or hypoxia were observed and no worsening of objective clinical signs occurred with advancement to the next- higher challenge dose.
- Isolated malaise, dizziness, abdominal cramps, nausea/vomiting, and signs of anxiety were recorded as localized reactions.

Systemic Reaction was defined as:

- sudden drop in systolic blood pressure as a single symptom; or
- generalized hives/flushing, angioedema, throat, lower respiratory, cardiovascular, gastrointestinal symptoms consistent with mast cell degranulation and involving at least two different systems, as commonly defined [10].

## Results

Sixty-six patients were identified who had been treated by sublingual immunotherapy for either systemic or localized reactions to shrimp and other crustaceans. All subjects fulfilled the criteria for diagnosis: history of reaction upon exposure consistent with immediate type (Coombs I) reaction; positive skin tests; positive shrimp IgE. Distribution of shrimp IgE concentrations and sizes of positive shrimp reactions to skin prick test are presented in Table 1. Prior to initiation of treatment, seventeen patients (25%) had had oral challenges to confirm the diagnosis of shrimp allergy (Table 1). Demographics and relevant comorbidities were consistent with those of the atopic predisposition (Table 2). Sublingual immunotherapy with five-fold serially diluted mixtures was initiated at 6.4, 64, 160 or 320 ng/dose and was gradually escalated to target dose of 0.5 mg/dose three times a day. The sublingual immunotherapy course ranged from 5 to 72 months (average: 51 months). When length of immunotherapy exceeded the intended maximum of 48 months, the target dose of 0.5 mg protein per dose was maintained until the date of the challenge.

**Table 1:** Demographic, clinical and immunological fetures of study subjects

Age (y)	Patients	Systemic event	Local event	Baseline challenge ( +)
6–18:	45	35	10	6
19–39:	17	14	3	11
40–65:	4	4		–
Total:	66	53 (80%)	13 (20%)	17 (25%)

Sex	
Female:	45 (68%)
Male:	21 (32%)

	Systemic event	Local event	Baseline challenge ( +)
sIgE range (kU/L)			
0.11–4.9:	3	2	2
5–19.9:	11	6	6
20–59.9:	21	4	6
60–99.9:	10	1	3
100–299.9:	5	–	–
> 300:	3	–	–
sIgE percent of total IgE			
0.1–0.9%:	–	–	–
1–2.9%:	–	2	–
3–4.9%:	2	5	2
5–9.9%:	6	2	5
10–19%:	24	1	6
20–40%:	12	3	4
> 40%:	9	-	–
Shrimp skin test vs. Histamine			
0.6x–1x:	12	6	6
1.1x–2x:	19	3	8
> 2x:	22	4	3

Age: age at the time of review

Systemic Event: number of subjects whose presentation was with a history of systemic reaction upon exposure

Local Event: number of subjects whose presentation was with a history of isolated symptom, or combination of symptoms from different systems whose localized nature and limited extent would not have required use of epinephrine

Baseline Challenge ( +): number of subjects in whom diagnosis of shrimp allergy with manifestations of systemic nature confirmed by oral challenge prior to initiation of sublingual immunotherapy

sIgE range: baseline sIgE on presentation. Samples that yielded values > 100 kU/L were diluted 1:10, re-tested, and the resulting concentration was corrected for 1:10 dilution

sIgE percent of total IgE: Shrimp IgE expressed as a percentage of Total IgE

Skin Test vs. Histamine: size of Shrimp skin prick test reaction (wheal’s maximal diameter) compared to Histamine control

**Table 2:** Baseline characteristics of study subjects who underwent shrimp challenges, and outcome of the original challenge

		Passed challenge	Localized symptoms		Passed challenge			Localized symptoms
Total challenged: 18		11	7	Age:				
Male:	7 (39%):	4	3	6–18:	6 (33%):	4		2
Female:	11 (61%):	7	4	19–61:	12 (67%):	7		5
History of presenting reaction:				Other food allergies:				
Systemic:	12 (68%):	7	5	Mollusk:	10		6	4
Localized:	6 (31%):	4	2	Fish:	4:		4	0
				Peanut:	2:		1	1
Other allergic diagnoses:				Tree nut:	1:		1	0
Asthma:	9 (50%):	5	4	No other:	4:		3	1
Rhinitis:	16 (88%):	10	6					
Atopic dermatitis:				Comorbidities:				
-Present:	3 (16%):	2	1	GERD:	5:		4	1
-History of:	13 (72%):	9	4	Thyroiditis:	2:		2	0

**Fig. 1:**
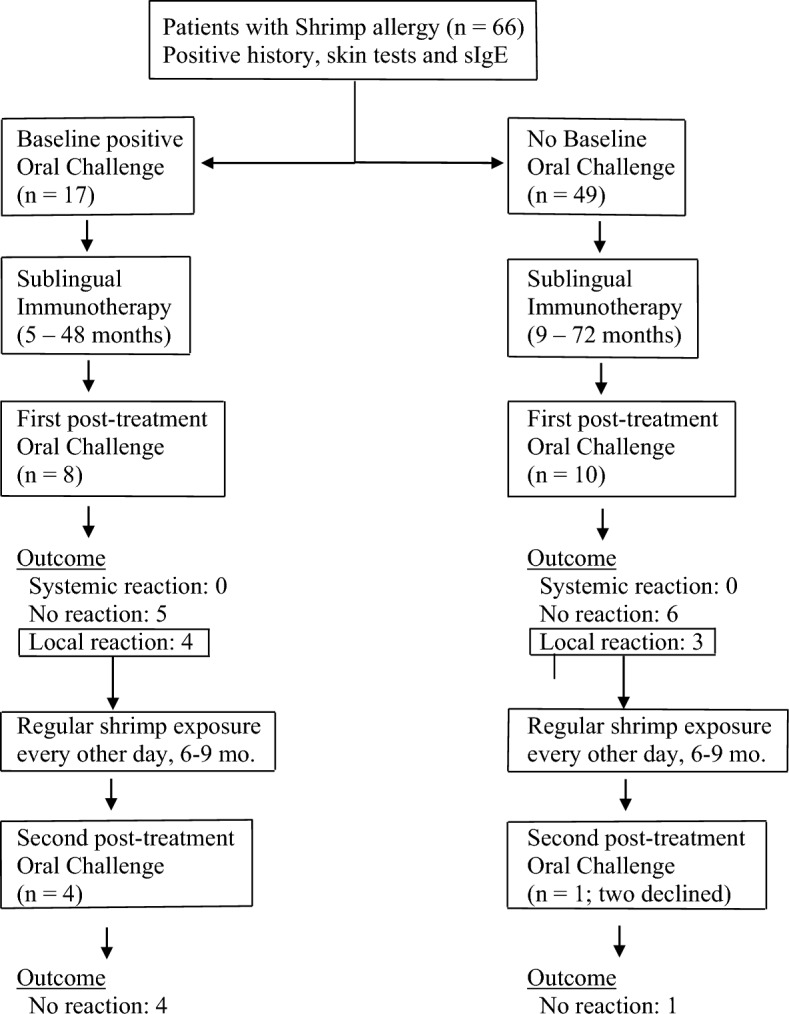
Patients with shrimp allergy were treated with sublingual immunotherapy and challenged at the end of immunotherapy treatment to cumulative doses of 81.2–148.2 g of lightly boiled shrimp over a period of 4 h. Patients with localized symptoms upon challenge were placed on 12–20 g every other day and re-challenged after 6–9 months of regular shrimp exposure

Eighteen patients underwent shrimp oral challenge. Twelve of the eighteen patients (66%) who were challenged after immunotherapy had originally presented with systemic reactions to crustaceans by history. Eight of them had had previous oral challenge(s) for shrimp which had resulted in systemic reactions, i.e. reactions originating in more than one system and associated changes in vital signs (Fig. 1).

No systemic reactions occurred during or after challenges; no patient required epinephrine (Table 2). Tolerance of target dose of 42 g or more was achieved in 11 patients (61%), with cumulative amount of 81.2 g for 5 patients, and cumulative amount varying from 106.2 to 148.2 g for six. Seven of these patients had originally presented with systemic reactions to crustaceans. Patients who passed the oral challenge were advised to consume shrimp and other crustaceans on a regular basis, one-two servings two–three times per week (Fig. 1). Since shrimp boiling time for the challenge was kept at minimum, variations in boiling time in post-challenge exposures did not appear to have an impact on tolerance as long as boiling time exceeded 60 s, according to instructions upon discharge.

Seven patients (38%) developed one or more of the following localized reactions: oral itching, nasal symptoms, localized perioral hives, localized hives at pressure points, nausea, vomiting, abdominal pain upon exposure to a cumulative amount of 39.2–148.2 g shrimp during the 4 h of the challenge. Five of these patients had originally presented with systemic reactions to crustaceans (Table 2).

Five of the seven patients who developed localized symptoms during the challenge were subsequently placed on routine exposure to 12–20 g of shrimp (two medium-size Gulf Shrimps or one Jumbo Shrimp) every other day with a view to repeating the challenge (Fig. 1). Two patients who experienced localized symptoms during challenge elected to continue sublingual immunotherapy but declined regular every-other-day intake of 12–20 g shrimp because at that time they decided against introduction of crustaceans to their routine diet; on follow up, they were satisfied with the outcome of the first challenge and wished to continue immunotherapy without having to undergo a repeat challenge. Repeat challenges were performed at 6–9 months of regular every-other-day consumption. On repeat challenge, all five patients who had followed regular every-other-day consumption of 12–20 g shrimp tolerated the procedure to target doses, which ranged from 42 and 67 g (cumulative amount 81.2 and 106.2 g respectively), without symptoms (Fig. 1). In summary:

- on 1st attempt, 11 patients (61%) passed and 7 patients (39%) experienced localized reactions
- on 2nd attempt, 5 patients (100%) passed.

On routine follow-up, none of the patients who passed the original or the repeat challenge reported any adverse reactions with regular intake of shrimp at the prescribed amounts or with larger amounts of other crustaceans—lobster, crab, crayfish included—consumed with meals.

## Discussion

The diagnosis of an IgE-mediated permanent food allergy is a life-changing event for patients and their families. The risk of a systemic reaction upon exposure and the possibility of anaphylaxis makes a life-threatening event out of any accidental exposure even when minute amounts of protein are involved or, especially in the case of crustaceans, when aerosolized particles are likely to be inhaled. Furthermore, each systemic episode is an entirely independent event which may turn out more severe than previous ones [1, 2, 11]. Adding to the uncertainty of systemic reactions due to food allergies, there is no diagnostic modality that would predict severity of reaction: skin tests and immunological assays for IgE antibody levels only predict likelihood of reaction and not severity [12]. The unpredictability of each reaction and the possibility of a fatal outcome places a constant burden on affected people who live under the threat of a sudden systemic reaction on practically every encounter, no matter how trivial, with food. Further complicating things, information on ingredients lists of manufactured foods is often misleading or erratic, and personnel involved in the preparation and serving of meals may lack access to relevant information. In light of these facts, long-term continuous control of immune responses to food allergens is a pressing necessity. Allergen-specific immunotherapy is the only disease-modifying modality for food allergy. Its implementation in a feasible, efficient, cost-effective, and safe way is the mainstay of long-term management [13]. With the present report, sublingual immunotherapy is presented, for the first time, as a useful modality in the long-term management of allergy to crustaceans. Administration of sublingual immunotherapy at home three times a day was tolerated by all patients and compliance remained satisfactory during the treatment period. During challenges, no systemic reactions were observed. Subsequent regular exposure to shrimp every other day was also tolerated without problems. Patients who remained asymptomatic during challenge, as well as patients who developed symptoms of localized nature with their first challenge and were placed on a limited-exposure protocol of 15–20 g every other day, have not developed symptoms with subsequent exposures to larger amounts of crustaceans. Consumption of shrimp at standard-serving (3 oz.) was not associated with symptoms on follow-up in either group.

The mechanisms of tolerance induction through sublingual immunotherapy have been extensively studied for long, but several of its long-term aspects are yet to be delineated [14]. Sublingual immunotherapy is user-friendly, inexpensive, free of significant complications, and is characterized by compliance levels superior to those of other forms of immunotherapy for allergens [15].

Regarding the long-term sustained outcome of desensitization, little is known and is mostly derived from experience with other food desensitization protocols. Sublingual immunotherapy for peanut allergy has been shown to result in decreased peanut-specific basophil activation and skin prick reactivity, as well as other parameters of IgE-effected sensitivity [16]. Upon discontinuation of sublingual immunotherapy, however, less than 11% of followed subjects had achieved sustained unresponsiveness [16]. Since sustained-unresponsiveness studies cannot be pursued in a private-practice setting, this issue will have to be addressed by larger-scale research projects. The patients of this cohort have all been advised to continue exposure to shrimp indefinitely at a minimum dose of 20 g every other day. As of the time of writing this report, no adverse events have been reported following regular intake of said amounts or with consumption of usual servings of crustaceans.

A pertinent feature of the present study is the assessment of symptoms and their evaluation in regard to their characterization as systemic or not. The resulting decision to continue with the challenge versus halting it was of major importance for the outcome of the challenges. By consensus, the emergence of a second symptom from a different system calls for an obligatory definition of the reaction at hand as an immune response of systemic nature [2, 10, 11]. In view of the risk for anaphylaxis, such a development would have led to cessation of the challenge and its assessment as a failure. For certain symptoms, however, a departure from the literary application of these criteria practice may be necessary, if these symptoms can be safely attributed to non-immune events, especially anxiety-related responses. In our study, certain symptoms were characterized as localized events even if they occurred along with other localized symptoms from a different system. Specific criteria for such an assessment were applied. Symptoms were termed as localized if: (i) they could be directly and unequivocally ascribed to anxiety, (ii) their severity had no measurable equivalent in objective parameters, (iii) increasing challenge doses led to no worsening of any other symptom and no escalation of the reaction was observed. The highly variable, subjective, and heterogenous nature of malaise, dizziness, nausea/vomiting, abdominal cramps, anxiety, tachycardia, and flushing is to be considered before the clinician in charge of the challenge arrives at the conclusion that mast cell degranulation of systemic magnitude has occurred. Without this modification and careful evaluation of symptoms within their context and timeliness, the rate of falsely-assessed failed food challenges is likely to be over-appreciated to the detriment of the patient’s interest.

A limitation of this study is that the challenges reported were all conducted with shrimp and this experience does not reflect established outcomes regarding other crustaceans, even though on follow up patients reported tolerance to other crustaceans. There is also a certain possibility that the tolerance that is demonstrated here may be limited to the three shrimp species that were exclusively used in the challenges. Furthermore, tropomyosin component testing was not performed and sensitization to Pen a 1 versus other shrimp allergens was not assessed [17]. Cross-reactivity with dust mite antigens was not studied either.

Fifty-three (80%) of the shrimp-allergic patients of the study had a history of a systemic reaction to shrimp, which was confirmed in 17 (25%) by baseline oral challenge. However, among the patients who underwent a post-treatment challenge, 12 patients (68%) had a history of systemic reactions and only 8 patients (44%) also had a positive oral shrimp baseline challenge before initiation of treatment. These discrepancies reflect a relatively higher willingness among patients with a history of localized-nature reactions to undergo a challenge, as well as for providers to order one.

A significant limitation of this study was the lack of pre-treatment baseline oral challenges for shrimp for 49 (75%) subjects. In a prospective study, routine baseline oral challenges would have been the standard of diagnosis. This drawback is inherent to the conditions of this study which was conducted in the setting of a private practice. The safety of the diagnosis of shrimp allergy for 75% subjects was based on the combination of the following positive findings: (i) all patients had been assessed by at least two Board certified Allergists from different practices, all of whom confirmed either the diagnosis of a systemic response, or a risk for a response serious enough to necessitate presription of Epinephrine and specific diet measures; (ii) all patients had positive shrimp skin tests performed by at least two different Allergists; (iii) by the time the post-treatment challenge was attempted, all patients had positive shrimp IgEs on at least 6 different occasions, several months apart, performed by different laboratories. The specificity of these combined data, although not as valid as a baseline oral challenge, was considered satisfactory for the purpose of treatment.

## Conclusions

Desensitization to shrimp by sublingual immunotherapy over a period of 5–72 months is assessed as a safe and effective treatment modality in the chronic management of allergy to crustacean. Tolerance to exposure is achieved and maintained while regular administration of modest amounts of shrimp continues. Whether the immune modification induced by sublingual immunotherapy is permanent resulting in sustained tolerance, or the achieved degree of desensitization depends on regular exposure is not known; therefore, following oral challenge, regular consumption three-four times per week is recommended.

## Data Availability

Study data are available at the Allergy Associates of La Crosse.

## References

[1] Sampson HA, Aceves S, Bock SA (2014). Food Allergy: a practice parameter update. J Allergy Clin Immunol.

[2] Muraro A, Halken S, Arshad SH, EAACI Food Allergy and Anaphylaxis Guidelines Group (2014). EAACI food allergy and anaphylaxis guidelines: primary prevention of food allergy. Allergy.

[3] Mansfield L (2006). Successful oral desensitization for systemic peanut allergy. Ann Allergy Asthma Immunol.

[4] Nelson HS, Lahr J, Rule R (1997). Treatment of anaphylactic sensitivity to peanuts by immunotherapy with injections of aqueous peanut extract. J Allergy Clin Immunol.

[5] Vickery BP, Vereda A, Casale TB (2018). AR 101 oral immunotherapy for peanut allergy. PALISADE Group of Clinical Investigators. NEJM.

[6] Kim EH, Yang L, Ye P (2019). Long-term sublingual immunotherapy for peanut allergy in children: clinical and immunological evidence of desensitization. J Allergy Clin Immunol.

[7] Wai CY, Leung PS (2022). Emerging approaches in the diagnosis and therapy in shellfish allergy. Curr Opin Allergy Clin Immunol.

[8] Wai CY, Leung NY, Hou Chou K (2020). Overcoming shellfish allergy: How far have we come?. Int J Mol Sci.

[9] Cortellini G, Spandolini I, Santucci A (2011). Improvement of shrimp allergy after sublingual immunotherapy for house dust mites: a case report. Eur Ann Allergy Clin Immunol.

[10] Lieberman P, Nicklas RA, Randolph C (2015). Anaphylaxis – a practice parameter update 2015. Ann Allergy Asthma Immunol.

[11] Sampson HA, Muñoz-Furlong A, Campbell RL (2006). Second symposium on the definition and management of anaphylaxis: summary, report—Second National Institutes of Allergy and Infectious Diseases/Food Allergy and Anaphylaxis Network symposium. J Allergy Clin Immunol.

[12] Ochfeld EN, Makhija M (2019). In vitro testing for allergic and immunological diseases. Allergy Asthma Proc.

[13] Lei DK, Saltoun CA (2019). Allergen immunotherapy: definition, indications, and reactions. Allergy Asthma Proc.

[14] Allam JP, Novak N (2014). Immunological mechanisms of sublingual immunotherapy. Curr Opin Allergy Clin Immunol.

[15] Okubo K, Izuhara K (2018). The status of sublingual immunotherapy in the treatment of allergic diseases. Allergol Int.

[16] Burks AW, Wood RA, Jones SM (2015). Sublingual immunotherapy for peanut allergy: long-term follow-up of a randomized multicenter trial. J Allergy Clin Immunol.

[17] Gámez C, Sánchez-García S, Ibáñez MD (2011). Tropomyosin IgE-positive results are a good predictor of shrimp allergy. Allergy.

